# Medical Students’ Reflections on Cultural Uncertainty During an International Global Health Program: A Qualitative Study

**DOI:** 10.1007/s40670-026-02668-w

**Published:** 2026-02-20

**Authors:** Theodore Joseph Miller, Julia Faust, William Hennrikus, Paige Richards, Carly Brogan, Katherine Wehrung, N. Ben Fredrick, Eileen Hennrikus

**Affiliations:** 1 The Pennsylvania State College of Medicine, 72 University Manor East, Hershey, PA 17033 USA; 2 Department of Medicine, Penn State Health Medical Center, Hershey, PA 17033 USA; 3 The Pennsylvania State College of Medicine, Global Health Center, Hershey, PA 17033 3 USA

**Keywords:** Global health, Cultural humility, Professional identity, Humanistic care, Medical education, Curricula

## Abstract

**Background:**

More than 40% of United States medical schools offer global health curricula designed to enhance students’ cultural awareness by exposing them to diverse populations, health inequities, and situations of systemic and interpersonal uncertainty. This study examined how medical students described changes in values, beliefs, and professional perspectives during immersive global health experiences within a structured global health program.

**Methods:**

We analyzed reflective journals from 52 Penn State College of Medicine students enrolled in the Global Health Scholars Pathway (GHSP), a longitudinal program with integrated lectures and a required international clinical elective. Students completed month-long placements at one of seven international sites (Ghana, Nepal, Vietnam, Zambia, Australia, Brazil, Ecuador). Weekly journal prompts performed at each site trip focused on experiences of intercultural uncertainty and foreignness. Three independent screeners verified completeness, and two reviewers conducted iterative, consensus-based thematic analysis informed by grounded theory principles.

**Results:**

Student reflections described responses to cultural difference across nine descriptive categories - extrinsic characteristics, language barriers, core beliefs, emotional attitudes, cultural engagement, intracultural relatedness, intrapersonal awareness, career growth, and observation & inquiry. These were then synthesized into three overarching thematic domains: Cultural Humility, Intrapersonal Resilience, and Realignment of Medical Motivations and Perspectives.

**Conclusions:**

Participation in the GHSP created conditions that supported reflection on intercultural uncertainty and professional values. Students described increased adaptability, emotional awareness, and reconsideration of professional priorities. Findings suggest that immersive global health curricula with structured reflection may support the development of culturally responsive and reflective clinicians. Future studies should examine transferability across sites and longer-term impacts on clinical practice.

## Introduction

Global health education has become an integral component of undergraduate medical training in the United States. Over the past decade, the proportion of allopathic medical schools offering structured global health programs has risen substantially from 24% to 42%, reflecting growing recognition within medical education that exposure to diverse health systems and sociocultural contexts holds relevance for supporting students’ professional and academic development [1, 2]. This growth also mirrors student demand and educators’ recognition that global health experiences may cultivate adaptability, empathy, and cultural competence. Despite this expansion, global health education modalities remain variable in curricular design, approaches to educational assessment, and ethical priorities [3].

To promote consistency and accountability, the Consortium of Universities for Global Health (CUGH) established a comprehensive competency framework encompassing 52 competencies across 14 domains [4]. The Penn State College of Medicine Global Health Scholars Program (GHSP) is a longitudinal educational program designed to support medical students’ development across several of these domains, particularly Domain 3 (Social and Environmental Determinants of Health) and Domain 10 (Sociocultural and Political Awareness). While the program includes preparatory coursework and lectures addressing how governmental, private, and non-governmental organizations influence global health and development, as well as how sustainable development goals (SDGs) and health, environmental, and poverty metrics guide global health agendas, students’ reflections mainly addressed sociocultural experiences. As such, this analysis focuses on sociocultural dimensions of global health engagement, with political awareness and power dynamics representing important areas for future curricular and analytic development.

The GHSP curriculum culminates in a required international clinical experience accompanied by structured reflective journaling during the visit. This reflective component is supported by physician mentors with the intention of guiding students in articulating their international experiences with ethical engagement, intercultural communication, and professional growth, along with how they have translated classroom-based learning into practice. These reflective capacities are increasingly recognized as important elements of physician preparation for navigating diverse health systems with humility and respect [5]. Designed to enhance students’ introspection and collaborative dialogue on their various clinical challenges and moments of sociocultural uncertainty, discussions with physician site leaders provide additional insight and perspectives on immersion experiences. However, while mentorship contextualizes the reflective environment of the program, this study focuses specifically on students’ written reflective journals; specific mentorship interactions and discussions were not independently analyzed.

### Transformative Learning as an Analytic Framework

As medical students engage in international service learning, research suggests that transformative learning experiences often occur [6]. Defined as learning that occurs when individuals are exposed to disorienting situations and are challenged with reforming past meaning with new frames of reference, exposure to unfamiliar cultural norms, alternative models of care, and navigation of resource constraints can prompt medical learners to examine previously unchallenged assumptions about medical institutions and “transform” their prior cognitive schemas of clinical care [6, 7]. Such experiences also have been suggested as encouraging opportunities for professional identity formation (PIF) and reflective “meaning-making” where recognition of personal biases, motivations, self-concepts, and knowledge limitations emerge and change [8]. Within this lens of transformative learning theory, the goals of the GHSP are intentionally operationalized to structure medical students’ learning with conditions of ambiguity, discomfort, and uncertainty in global clinical settings.

**Fig. 1:**
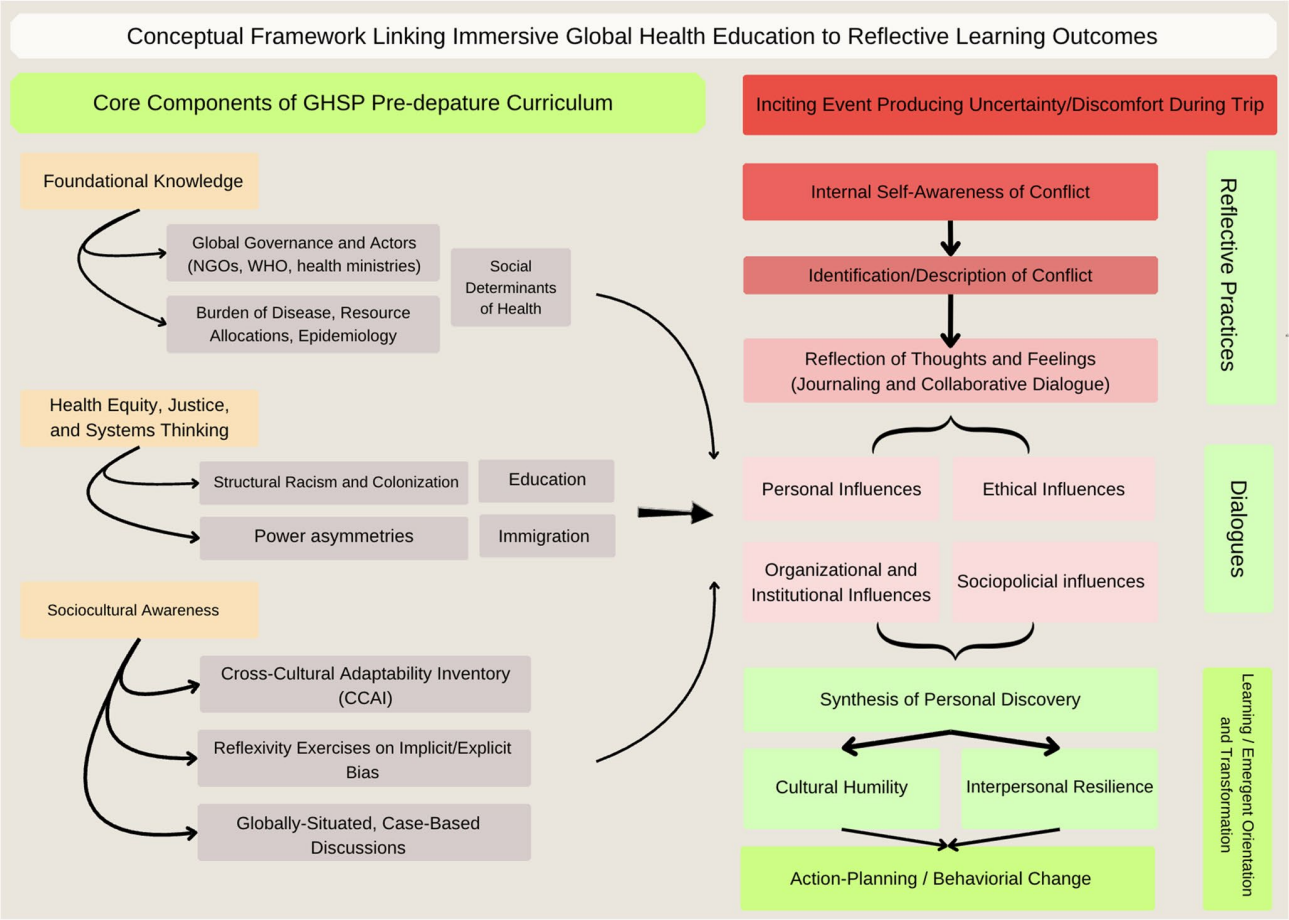
Conceptual Framework Linking Immersive Global Health Education to Reflective Learning Outcomes. Framework depicting how pre-departure curricular components prepare students for reflective engagement with uncertainty during global health immersion, leading through structured reflection and dialogue to cultural humility, interpersonal resilience, and action-oriented professional growth

As seen in Fig. 1, our conceptual framework illustrates how immersive global health experiences within a structured educational program may support reflective learning processes in medical students. Preparatory training, international clinical immersion, and structured reflective journaling create opportunities for students to navigate disorienting experiences characterized by cultural difference, ambiguity, and uncertainty at their respective sites. Through reflective meaning-making, these experiences may engage transformative learning processes that contribute to professional identity formation. Cultural humility, intrapersonal resilience, and realignment of professional motivations are also represented as possible emergent orientations described within reflective narratives, rather than discrete competencies or measured outcomes.

### Thematic Conceptual Framework and Study Aims

 Guided by Transformative Learning Theory, this study applies a thematic conceptual framework to examine how immersive global health experiences are described within students’ reflective writing [6, 7]. We analyze reflective narratives generated in disorienting clinical and cultural contexts to explore how students reported processes of meaning-making related to professional identity formation and orientations towards cultural humility, intrapersonal resilience, and professional motivations.

Specifically, we sought to answer:

1. How do students describe and interpret experiences of intercultural uncertainty during participation in GHSP?
2. In what ways do students reflect on cultural humility and intrapersonal resilience within their written narratives?
3. How do students describe shifts in perspective related to their motivations and understanding of medical practice?

Despite growing interest in global health education and the establishment of competency frameworks, gaps remain in understanding how specific curricular structures may support reflective learning processes [9, 10]. Our study directs attention toward moments of uncertainty, discomfort, and perspective shifts within students’ reflective narratives. By grounding our analysis of students’ narratives in this way, we contribute to the educational discourse on how self-examination through global health immersion trips may alter medical students’ understanding of different cultures and approach to unfamiliar environments. The outcomes of GHSP underscore the need for more explicit alignment between global health programming and opportunities for structured student reflection around moments of uncertainty to support students’ development of a more open and inclusive worldview.

## Materials and methods

### Study Design - Qualitative Analysis of Journal Responses

Our study employed a descriptive, qualitative design approach that utilized inductive principles based on grounded theory methodology. Centered on simultaneously analyzing interview observations and identifying emerging concepts and theories, this open-ended approach allowed for the comprehensive capture of themes, events, and emotional responses underlying participants’ global health experiences [11]. By prioritizing participants’ own language and perspectives, we sought to contextualize their reflections within the social and cultural frameworks of their specific host environments. Written narratives were examined with a priority to identify meaningful patterns related to intercultural uncertainty, adaptation, and personal growth throughout the duration of each trip.

This study was not designed to compare sites as discrete analytic units. Instead, sites were treated as contextual conditions informing students’ experiences. While site-specific differences may influence how cultural uncertainty is encountered, the analytic focus was on shared processes across settings rather than comparative site analysis.

### Participants

A total of 52 1st year medical students from Penn State College of Medicine participated in the Global Health Scholars Pathway (GHSP), a longitudinal program throughout all four years with integrated immersive international, month-long host-site trips designed to promote experiential learning and cultural competency. Students’ global health experiences ranged across many diverse international sites, including Ghana, Brazil, Ecuador, Australia, Nepal, Vietnam, and Zambia. Each participant was involved to some varying degree in community health initiatives and clinical care within their respective host sites and had established longitudinal relationships with their host environments aimed at enriching their intrinsic schema of global health measures and disparities.

All students participating in GHSP were required to report evolving values, conflicts, and perceptions through journal assessments and entries. Journal entries prompted students to consider three different themes when answering: their experiences with intercultural uncertainty and connections, their experiences with foreign novelty, and their identification of value incongruity. Questions were presented to students under two different subject titles: (1) Intercultural Uncertainty and (2) Feeling Foreign - Being The Other. Trips consisted of one month-long period during the summer with journals collected at the conclusion. Three independent screeners reviewed all journal submissions, and responses that were illegible, incomplete, or empty were excluded. This resulted in 50 viable entries for the “Intercultural Uncertainty” subject title and 52 for “Feeling Foreign—Being the Other” subject title.

Representation of responses reflects multiple individualized backgrounds, including extensive geographic diversity and baseline interactions with international countries prior to initiation of GHSP; this accounts, in part, for the potentially variable situational differences experienced by students within their respective journal entries.

### Data Collection

Medical students at Penn State College of Medicine completed a series of structured reflective journal prompts designed to explore their perceptions of their individual GHSP experiences and their impressions of their host community’s cultural values. Each prompt aimed to elicit reflections on moments of uncertainty, ambiguity, and value conflict encountered during their time abroad, along with self-evaluation on how each student responded to discrepancies between their beliefs, emotions, and values with those of their site. Students were tasked with compiling responses during their GHSP trip, conducting weekly retrospection on the events they experienced at their site and annotating their responses.

Journal prompts were arranged into two respective subjects, and response data were collected based on multiple assigned survey questions listed below in Table 1. These prompts were intentionally open-ended and experiential rather than psychometrically validated, consistent with grounded theory’s emphasis on eliciting participant-generated meaning rather than measuring predefined constructs. Prompts were designed to function as sensitizing devices, encouraging reflection on uncertainty, foreignness, and value incongruity without constraining students’ responses to predetermined categories.

We acknowledge that these journal prompts have not undergone formal validation. As such, responses may reflect variability in interpretive depth and writing style. However, this variability was considered analytically valuable, allowing unanticipated processes and perspectives to emerge inductively from the data.

**Table 1:** Journal prompts for global health students

Subject:	Questions
“Intercultural Uncertainty”	*Q1: “What: Describe objectively an experience when you encountered intercultural uncertainty. Who was present? What was the setting? What happened? This could be as simple as not knowing how to barter*,* whether or not to leave a tip*,* or determining how to get from Point A to Point B. Or it may be more substantial*,* such as uncertainty about whether you have offended someone or hurt their feelings. How did you respond to the uncertainty? What decisions did you make?”* *Q2: “So What: Why do you think this instance stuck out to you? How does uncertainty affect you? What about your decision-making?”* *Q3: “Now What: What did this encounter teach you about how you currently manage uncertainty? What do you want to affirm in your approach? What might you do differently next time?”*
“Feeling Foreign - Being The Other”	*Q1: “What: Describe your feelings of being other and illustrate this with examples from your experiences on the trip. How did you overcome feelings of being “Other”? Who or what helped you with this?”* *Q2: “So What? How are you managing the differences you are encountering? How does being the “Other” impact your willingness to be involved at times?”* *Q3: “Now What: Consider how being a minority group member in the US might lead to challenges when interacting with a US clinic or hospital staff. What problems might arise as a result? What else do you need to learn about this topic?”*

Journal prompts adapted from the Global Health Scholars Program at Penn State College of Medicine that were designed to promote MS1 student reflection on intercultural awareness, uncertainty, and adaptive growth during global health experiences

### Qualitative Data Analysis

We employed a constructivist grounded theory approach to explore how medical students make meaning of global health engagements. Consistent with grounded theory principles, data collection and analysis occurred iteratively, allowing analytic insights to inform ongoing coding decisions and theoretical development. Grounded theory principles were operationalized through concurrent data familiarization and coding, constant comparison within and across journal entries, iterative refinement of codes through multiple analytic cycles, and theoretical integration of codes into higher-order conceptual categories, Rather than treating themes as discrete endpoints, analysis emphasized relationships among codes to generate an explanatory framework describing how students experienced and responded to cultural uncertainty during global health engagement.

Two primary coders independently conducted initial line-by-line and incident-by-incident coding of all journal entries. Codes were inductively generated, remained close to the participant’s language, and were framed as actions or processes where possible (e.g., navigating language discordance, questioning assumptions, observing cultural norms). During this phase, an open coding framework was applied, where categories emerged directly from the data instead of pre-set codes of theories. This produced an initial, tentative set of descriptive and process-oriented codes, generated from emerging themes from the journal data and condensed into a preliminary “codebook” that highlighted commonalities between students’ GHSP experiences. This codebook acted as a template on which subsequent review cycles were performed.

Through focused coding, the coders identified the most salient and frequently occurring codes. Using constant comparative methods, codes were compared across participants, across journal prompts, and across international sites. Each reviewer provided supportive quotes to introduce context and evidence for the application of each code within the data. Analytic memos documented evolving code definitions, relationships among codes, and emerging conceptual patterns. These memos informed iterative refinement of the codebook, including consolidation, modification, or elimination of codes across successive analytic cycles. Coding disagreements were resolved through structured discussion and consensus rather than calculation of statistical interrater reliability, consistent with constructionist grounded theory methodology. When discrepancies arose, coders reviewed the relevant excerpts jointly, revised coding definitions, and refined the codebook accordingly. This process prioritized conceptual clarity and theoretical coherence over numerical agreement.

Multiple iterative review cycles of all journal entries were conducted on the journal’s two subjects to ensure the efficacy, accuracy, and reliability of our themes. Review cycles continued over four months, with the last iterative review leading to a reduced, yet more accurate final codebook that included a set of nine robust “codes” with strong thematic evidence and encompassed all significant themes generated from both journal subjects. These codes serve as conceptual anchors representing interrelated dimensions of students’ experiences. Table 2 outlines our finalized code set with examples from our journal analysis.

Codes were considered “robust” when they met three criteria: presence across a substantial proportion of participants (≥ 5 entries, or approximately 10% of journal entries), recurrence across multiple international sites, and conceptual richness, defined by multiple properties and variations identified through constant comparison. Frequency alone was not used as a determinant of analytic importance.

**Table 2:** Final codebook with journal examples

Medical Student Experiences With Cultural Uncertainty and Foreign Ambiguity		
Code name	Brief Description	Example Student Quote
Extrinsic Characteristics	Students identify their “otherness” as being tied to superficial characteristics	“Although I am Asian and share similarities to the people here, I have definitely felt the “other” frequently… we will often get stared at by patients and bystanders. It is very obvious that I am an other.”
Language Barriers	Students report that language/communication differences create additional strain on their ability to interact with and understand their environment	“At times, I felt “the Other” especially when several people in the group were speaking Vietnamese to each other and laughing and I couldn’t understand… The language barrier can be frustrating and hinders communication or my willingness to ask questions, especially that certain phrases don’t translate well from English to other languages”
Core Beliefs	Students describe discordance of their individual feelings, beliefs, or attitudes with that of their host country or community	“We were confused because everyone talked as if they were excited to play volleyball, but no one came except us. In a way we were upset and felt like we were lied to.”
Emotional Attitudes	Students explicitly describe uncertainty as relating to emotional considerations when interacting with their community	“As a gift for us when we were preparing to leave, they offered the three of us an entire butchered goat. This brought about uncertainty because we didn’t want to offend by denying their gift, but we both didn’t want to take such a large gift from a poorer family and probably wouldn’t be able to eat it all”
Cultural Engagement	Students cite engaging with new food, routines, apps, clothes, languages, or skills unique to their host’s community	“My first smoking ceremony was something that caused me great uncertainty… as the new clinic was being blessed, one of the Aboriginal community members came up to me and blessed me with leaves… faced with uncertainty, I decided to copy the behaviors of those around me”
Intracultural Relatedness	Students mention finding common ground between their perceived cultural, religious, or personal identity to that of their new site or environment	“In Australia as a white female, I blend in… On appearance, we are not as foreign. Our dress is similar. They also have similar patterns of social interaction.”
Intrapersonal Growth	Students cite a personal interaction, event, or process as providing them new insight or reflection into their moral, ethical, or personal beliefs	“If I could do anything differently, it would be to try to know more about a culture to help to know how to make similar tough decisions in the future.”
Career Growth	Students highlight an experience abroad as enhancing their perspective and knowledge pertaining to their career in medicine	“This is extremely relevant to my future practice of medicine as I will encounter people of different cultural backgrounds and practices. Each of these cultures has their own norms of practice that will be relevant to properly care for the patient.”
Observation & Inquiry	Students highlight tackling uncertainty with meaningful intent and investigation of their environment	“I want to learn more about initiatives in healthcare to prevent errors from happening and how healthcare professionals are trained to use medical interpreters”

Codes and illustrative student quotes derived from reflective journals of participants in the Global Health Scholars Program. The identified themes reflect the dynamic growth process across individual, intrapersonal, and professional domains that students’ experienced during the global health journey. The overarching themes emphasize adaptability, self-awareness, and cross-cultural learning

Additionally, we sought to ensure accurate consensus and replicability of the finalized codebook. An independent review of three randomized response sets of ten journal participants was conducted. Application of each “code” was conducted on each journal response, with lab members discussing their choice and achieving consensus when each party obtained the same, validated response. Continued revisions were made when applicable in events of coding disagreements, and this process persisted in randomized order until adequate interrater reliability was achieved.

The research team consisted of medical trainees and faculty with prior experience in global health education. The team’s positionality, including shared professional identity with participants and varying degrees of prior international exposure, was explicitly acknowledged throughout the analysis. Reflexive discussions were incorporated into coding meetings to examine how researchers’ assumptions about culture, medicine, and professional growth might shape interpretation of the data. Analytic memos documented these reflections and were revisited during theory development to minimize unexamined bias and enhance interpretive transparency.

## Results

### Global Health Engagements Fostered Themes of Humility, Resiliency, and Personal Goal Setting

Across GHSP programs, students described experiences of navigating unfamiliar cultural, social, and clinical environments. Journal reflections frequently addressed moments of discomfort and uncertainty, as well as subsequent changes in how students describe their emotional responses and professional priorities. Three recurring domains were identified through analysis: cultural humility, in global health engagements, intrapersonal resiliency against cultural uncertainty, and realignment of goal-centered medical motivations and perspectives. These domains were reflected across sites and prompts and were supported by multiple codes within the final codebook.

**Fig. 2:**
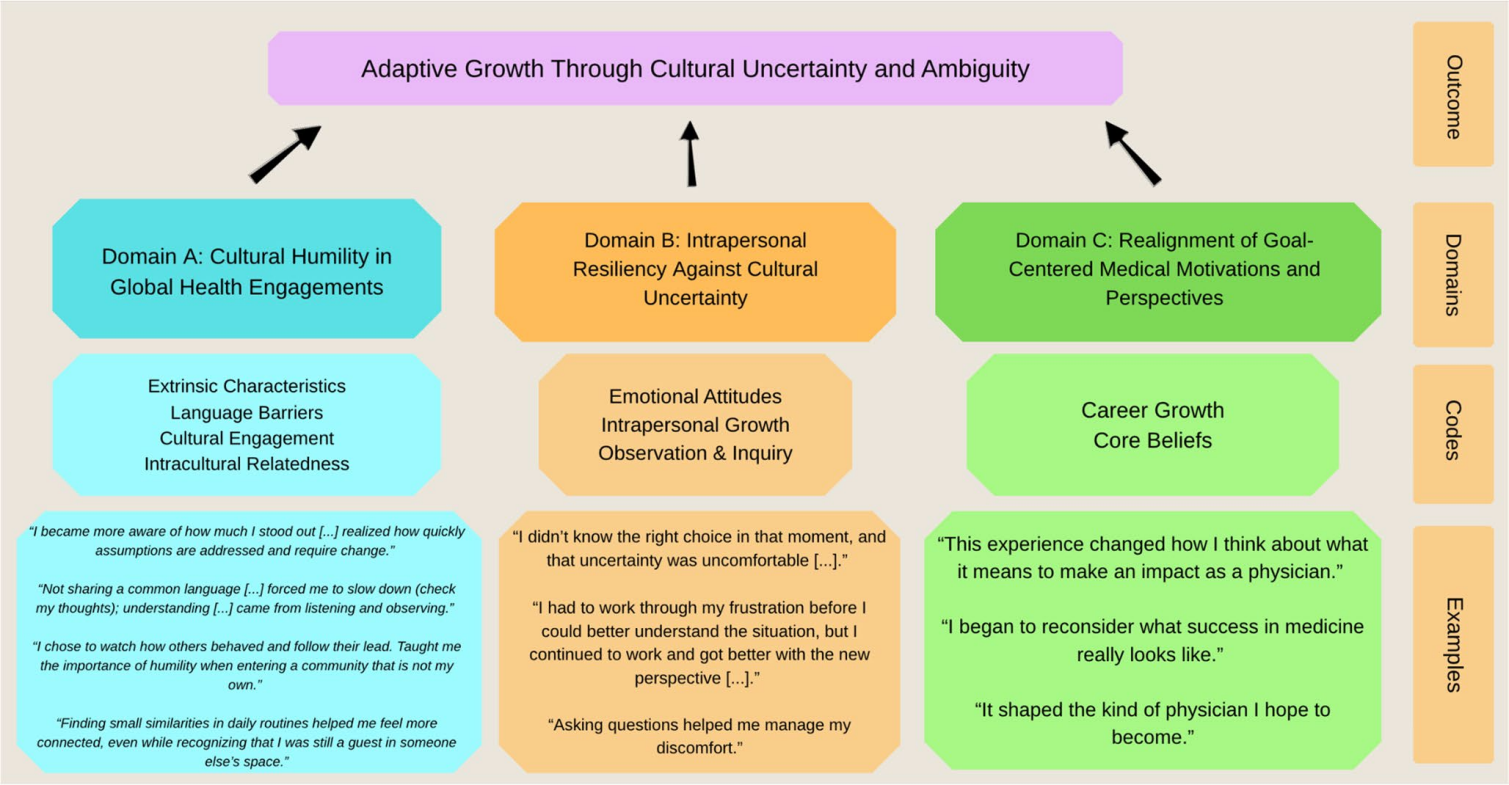
Thematic Map of Codes and Domains Identified in Students’ Reflective Narratives. Thematic map illustrating relationships between inductively derived codes and three overarching analytic domains: (**A**) Cultural Humility in Global Health Engagements, (**B**) Intrapersonal Resiliency Against Cultural Uncertainty, and (**C**) Realignment of Goal-Centered Medical Motivations and Perspectives. Quotations are provided to demonstrate analytic grounding and to highlight how students’ reflective narratives progress from external cultural encounters to internal adaptation and evolving professional identity

### Domain A: Cultural Humility in Global Health Engagements

Students frequently described encounters that prompted reflection on their assumptions, expectations, and roles as visiting trainees within unfamiliar cultural contexts. Codes supporting this domain included extrinsic characteristics, language barriers, cultural engagement, observation and inquiry, and intracultural relatedness.

“*I operate under the bias/assumption that most people have the same baseline education level as me which is clearly not always true… the doctor and I were teaching midwives and nurses how to do breast exams to detect cancer*,* however we realized they had little concept of cancer*.”

Others described realizing how deeply their training shaped their initial judgments about care practices. For example, one student noted discomfort observing non-biomedical approaches to treatment –.

“*Herbal medicine was big in the past because of the lack of access*,* but even with gaining access*,* people still really rely on herbal medicine*”.

Similarly, students described confusion or unease when encountering unfamiliar rituals integrated into clinical care.

“*I was confused by the incorporation of indigenous rituals before rounding on patients*,* but I eventually understood how important it was to them and how this could be used back home on wards”*.

Recognition of privilege and positionality also emerged in several reflections. One student wrote –.

“*I didn’t notice how much privilege I carried until I saw how differently patients responded to me versus other team members*”.

Students also described becoming aware of how their own norms shaped their interpretations of safety and professionalism. One student reflected on hospital infrastructure –.

“When *I saw areas of the hospital/OR that lacked hygiene and safety standards*,* I could not help but imagine all of the possible bad outcomes from these practices*.”

Later in the same reflection, the student notes a shift in how they approached these differences, saying –.

“*You learn to be comfortable with being uncomfortable finally*,* be open to these differences*,* why they may occur in different regions*,* and don’t see them as inferior to your way of doing things.*”

Several entries highlighted how direct engagement and inquiry facilitate reconsideration of initial reactions. One student described recognizing interpersonal norms they had previously viewed negatively –.

“*To me it’s an inconvenience*,* something I don’t want that they forced upon me. To them culturally it is a welcoming gift to display their hospitality to me very graciously*.”

Others reflected on differences in communication and interaction styles:

“*They explained how it was common for people to hug with greetings and stand closer together while talking*,* which they did not experience [with Americans] being more distant. I tried being warmer*”.

Across reflections, students described increased awareness of how their assumptions and training shaped their interpretations, followed by efforts to question or recalibrate these perspectives. Engagement in community activities, such as participating in rituals, adapting to social customs, or observing how cultural values are integrated into care, featured prominently in these accounts.

### Domain B: Intrapersonal Resiliency against Cultural Uncertainty

Students described numerous experiences that challenged their adaptability and emotional regulation during their global health immersions. Codes supporting this domain included core beliefs, emotional attitudes, and intrapersonal growth.

Early reflections often described feelings of uncertainty, frustration, or inadequacy. One student shared –.

*“I felt useless the first week*,* but I think that was due to having to navigate a new health system and environment than the U.S. I knew I just did not understand how things worked here*,* so I asked the consultants many questions”*.

Others described becoming more aware of how they personally respond to uncertainty stating,

“*I learned that I tend to stay away from the unknown and get very stressed in spontaneous situations.”*

Students frequently described changes in how they approached these moments over time. Several emphasized the importance of recognizing uncertainty rather than avoiding it –.

“*I learned I need to actively acknowledge intercultural uncertainty when I encounter them and not bury it under the rug. By actively noticing and making changes is how I can learn about other cultures and values*”.

Peer discussion and shared reflection were also cited as strategies for managing discomfort –.

“*I dealt with conflict of values by making an effort to recognize these disparities of values and slowly trying to adapt… talking about them with others was also helpful.*”

Adaptation was often described through concrete behavioral changes, such as adjusting to community practices –.

“*The main way I dealt with conflict in values was by understanding that cultural differences in values exist and by trying to adapt to what my host community values. For example*,* a simple change was fire orientation. Every gathering*,* elders started fires*,* and I adapted by following similar trends when meeting with friends*”.

Students also described reframing uncertainty more positively, with one noting –.

“*Everything always works out… it can be even be a bit fun to have no idea what you’re doing or where you’re going.”*

Across entries, students describe learning to remain flexible, tolerate ambiguity, and continue engaging despite discomfort, rather than resolving uncertainty entirely.

### Domain C: Realignment of Goal-Centered Medical Motivations and Perspectives

Many students described shifts in how they articulated their professional goals following their global health experiences. Codes supporting this domain included career growth, core beliefs, observation and inquiry, and intrapersonal growth.

Several students reflected on moving away from narrowly defined goals toward broader, relational, or service-oriented goals. One student wrote –.

“*This experience opened my eyes to my desire to be a resource and set of listening ears to my patients. I hope to be someone they can confide in.*”

Others emphasized advocacy and leadership as emerging priorities –.

“*In my future practice I will need to be an advocate for my patients… I feel I will set the culture and tone of the environment I work in*”.

Some students described identifying tangible needs within host communities that shaped their future intentions –.

“*I saw a dire need for ophthalmology equipment in Koforidua…and hope to apply for grant funding when I return*”.

Students also reflected on how experiences of being “the other” informed their future approach to patient care –.

“*All patients bring their unique backgrounds and experiences with them into the hospital and being comfortable with incorporating their values & beliefs into their medical care is important to building patient -physician trust & optimizing care*”.

Across reflections, students described reassessing what they valued in medicine, often emphasizing listening, relationship-building, and responsiveness to patients’ social and cultural contexts.

## Discussion

Our study explored how participation in the Global Health Scholars Program (GHSP) shaped multiple components of medical students’ professional identity formation. Students’ reflections also revealed growth across three interrelated domains: cultural humility, intrapersonal resiliency, and goal-centered motivation. Together, our findings highlight how immersive global health programs in medical education can promote reflection, adaptability, and a renewed sense of purpose in healthcare for student learners, along with increased competencies needed for success in residency.

### Cultural Humility: Learning Through Discomfort

Cultural humility, or the continual art of intrapersonal criticism and willing inquiry into adapting to unknowns, is a foundational competency for effective global health engagement [3]. Students described processes consistent with movement toward cultural humility, including recognition of assumptions, openness to alternative frameworks, and acknowledgement of limits to their knowledge. While students’ descriptions of discomfort-triggered reflection align with Mezirow’s transformative learning theory, cultural humility is reflected not in the experience of dissonance itself, but in students’ recognition of power, positionality, and the limits of their expertise [7, 12].

Identification of discordance soon followed realizations that students’ perceptions were based on Western medical standards and values. Specifically, students noted significant reframing of their mental models of healthcare and demonstrated respect for alternative modalities of care and system structures. Weekly journal reflections mirrored a prominent distinction between cultural competency and cultural humility, with students’ global health experiences suggesting that opportunities abroad extend learning past rote knowledge of other cultures into understanding their dynamic positionality in clinical care [12]. As students participated in community rituals and worked within unfamiliar health systems, they reported examples of this growth and noted deeper appreciations for how culture informs care. Our findings suggest that global health immersion may function as a disorienting dilemma, while movement toward cultural humility depends on whether reflection specifically addresses bias, power, and limitation [12]. This reinforces similar themes from prior global health education research in that dissonance and uncertainty can spark opportunities for self-awareness and empathy [13]. Global immersion, therefore, becomes not an exercise in knowledge acquisition, but in identity disruption.

### Intrapersonal Resiliency: Adapting To Uncertainty

Exposure to resource limitations, communication barriers, and role ambiguity challenged students’ emerging professional identities. Students reported concepts of intrapersonal resiliency, or the ability to adapt and maintain purpose amid uncertainty. Early fragility and frustration navigating these limitations evolved into adaptive confidence through reflective practice, aligning with Mezirow’s transformative learning theory, where disorienting dilemmas stimulate the restructuring of assumptions [7, 14, 15]. Students described choosing to lean into discomfort: tasting unfamiliar foods, adopting new greeting rituals, or questioning hierarchies. This progression reflects Mezirow’s theory of transformative learning, where reflection on disorienting experiences leads to new perspectives and behaviors [7, 14, 15].

Resilience reportedly emerged as an active process of integration rather than endurance and survival by students in their cultural environments. As students noted managing discomfort, whether through engaging in local customs or adjusting to unfamiliar clinical structures, they discussed becoming more flexible and confident with their decision-making schemas in foreign environments. Previous work shows that this form of reflective engagement with uncertainty can foster characteristics of empathy, adaptability, and professional maturity [6, 14, 15]. In this sense, humility and resilience may have functioned synergistically: humility opening students to new experiences, and resilience allowing them to remain engaged within them. Our results revealed that students’ varied experiences not only enriched aspects of cultural humility but also fostered intrapersonal resiliency through allowing actionable growth in moments of cultural uncertainty - skills that will be invaluable for future medical practice.

### Goal-Centered Motivation

Finally, students consistently reported a reframing of their motivations in medicine from personal achievement to collective responsibility. Realignment of students’ goal-centered motivations occurred repeatedly in journal responses, and we observed our cohort report that the additional context from their trips assisted with their engagement in new interests and skills. Early goals reported by students highlighted prioritization of technical skill acquisition and expansion of clinical knowledge. Post-trip reflections, however, revealed a shift in students’ motivations toward developing culturally competent care and health equity strategies. Several students reported taking concrete steps toward their new motivations, such as grant writing for clinic supplies and establishing new partnerships across hospital systems based on disparities they witnessed abroad. Global health, in this context, potentially ignited moral imagination and career shifts from students. This expanded students’ sense of obligation to underserved communities echoes prior findings that global engagement can deepen altruism and social accountability, which are necessary competencies for medical students before residency training [15, 16]. This evolution also aligns with self-determination theory’s assertion that intrinsic motivation grows through autonomy and connection to others [17].

By situating medicine within a broader international context, students reframed their purpose from individual achievement to collective social accountability - a transformation known to support physician well-being and resilience [18]. These reflections demonstrate how the realignment of goal-centered medical motivations and perspectives can be exercised through global health initiatives, along with how they can fasten students into future roles as empathetic, service-driven physicians who prioritize understanding, collaboration, and equity in all clinical encounters.

## Conclusion

Our immersive global health program (GHSP) has shown evidence of creating conditions for medical students that promote cultural humility, professional development, and intrapersonal resilience. We attribute these benefits as being connected to the structured operationalization of our global health program and its integrated opportunities for reflection at each site. Disorienting experiences through GHSP challenged the preconceived ethnographic assumptions of students, and we propose that through meaningful opportunities for reflection, students were able to address their discomfort and grow in both clinical and professional domains.

This qualitative analysis suggests that specific curricular structures, rather than immersion alone, may shape students perceived professional and ethical development during global health experiences. Across reflective journals, students consistently described structured journaling as a mechanism for identifying ethical tensions, discomfort, and uncertainty. Students also frequently attributed their most meaningful learning to community-engaged roles, particularly learning directly from host community members, and contrasted this with more passive observational experiences. Although variation across host sites limits the transferability of specific activities, the recurrence of these themes across journals indicates that other programs could benefit from prioritizing self-reflection and collaborative discussions during immersion events. Given the reliance on self-reported reflections, these findings reflect perceived educational impact rather than objectively measured outcomes. Nonetheless, our students’ perspectives suggest benefits of global health curricular designs that intentionally integrate structured reflection activities and discussions within their program through their potential role promoting medical students’ cultural humility and intrapersonal growth [19].

Findings from students’ reflective journals also emphasize the importance of distinguishing immersive global health education from voluntourism-oriented approaches. Voluntourism has been characterized by short-term, service-focused experiences that may prioritize learner exposure over community partnership and risk reinforcing inequities and hierarchical power dynamics [20]. In contrast, students in this cohort consistently described their most meaningful learning experiences as emerging from curricular elements that emphasized reciprocal engagement with community-defined learning roles in hospitals or clinics, along with opportunities to learn directly from host community members and having structured reflection opportunities rather than being passive observers. Although student participation occurred over a limited time frame, journals suggested that educational value was shaped less by duration than by this type of curriculum structure. While these findings reflect perceived educational impact and are not broadly generalizable, they suggest that, even in time-limited settings, global health curricula that is intentionally designed around reflection and community engagement may better support the development of professional, community-oriented medical learners [21, 22].

We recognize several limitations within our study that restrict the generalizability and face validity of our findings. Our GHSP participants were given novel journal prompts with no prior validation, and we could not confirm whether students strictly adhered to weekly journaling schedules. Observer bias may have influenced student responses, as all participants knew their reflections would be evaluated. This potentially leads to more socially desirable reporting with emphasis on altruism and cultural competency. Additionally, our analysis did not disaggregate themes by host site, limiting insight into how differences in clinical settings, community engagement, or local culture may have shaped individual experiences. This introduces challenges in assessing the transferability of findings to other global health contexts or programs. Future research could explore site-specific variations, incorporate voices from community perspectives on student engagement, and examine discrepancies between learners’ introspection and external observations. Longitudinal studies may also further clarify how immersive global health experiences influence professional development, residency selection, and sustained engagement in equity-focused work throughout medical careers.

## Data Availability

The data that support the findings of this study are available on request from the corresponding author, TJM. Restrictions apply to these data due to privacy and ethical considerations.
